# Disparities in Chronic Disease Among Canada’s Low-Income Populations

**Published:** 2009-09-15

**Authors:** Raymond Fang, Andrew Kmetic, John Millar, Lydia Drasic

**Affiliations:** British Columbia Provincial Health Services Authority; British Columbia Provincial Health Services Authority, Vancouver, British Columbia, Canada; British Columbia Provincial Health Services Authority, Vancouver, British Columbia, Canada; British Columbia Provincial Health Services Authority, Vancouver, British Columbia, Canada

## Abstract

**Introduction:**

Many studies have found inequities in health among income groups in Canada. We report the variations in the major chronic disease risks among low-income populations, by province of residence, as a proxy measure of social environment.

**Methods:**

We used estimates from the 2005 Canadian Community Health Survey to study residents who were aged 45 years or older and from the lowest income quintile nationally. Multivariate logistic regression was used to examine the relationship between province of residence and risk of chronic diseases.

**Results:**

British Columbia is the healthiest province overall but not in terms of its low-income residents, whereas Quebec's low-income residents are at the least risk for major chronic diseases. The significant differences in risk of hypertension, diabetes, and heart disease in favor of British Columbia over Quebec for the entire population disappear when considering only the low-income subset.

**Conclusion:**

Quebec's antipoverty strategy, formalized as law in 2002, has led to social and health care policies that appear to give its low-income residents advantages in chronic disease prevention. Our findings demonstrate that chronic disease prevalence is associated with investment in social supports to vulnerable populations.

## Introduction

Socioeconomic determinants of health and inequities in health outcomes have drawn increasing attention in recent years from academic and health care professionals as well as policy makers (1-6). Health inequities are unfair and avoidable differences in health status among populations. In Canada, a country with a publicly funded health care system, we still see that the lower people are in the socioeconomic hierarchy, the shorter their life expectancy (7) and the higher their risk of developing chronic diseases (8-10).

Health status is associated with behavior and with work and home environments, which are determined by a person's socioeconomic status. Thus, socioeconomic determinants are known as the "causes of the causes" of health (3). The socioeconomic determinants of health are not simply a measure of wealth but a synthesis of wealth, education, and social and physical environments.

The existence of excess chronic diseases in Canada's low-income population, compared with its high-income population, is amenable to policy interventions. Health conditions have a strong geographical dimension across the 10 provinces of Canada. In 2005, there were gaps in life expectancy at birth of 3.2 years and 2.6 years for men and women, respectively, between the province with the longest life expectancy, British Columbia, and the province with the shortest, Newfoundland and Labrador (11). These differences in life expectancies reflect provincial inequities in health across the nation.

Unfortunately, Canada has neither a national social support system nor a uniform health care policy to address health inequities among provinces. Instead, the 10 provinces have 10 different social and health systems, each with complex regulations. The resulting differences in social assistance and health services may differentially affect the quality of life and the health conditions of the low-income residents of different provinces.

Inequities in health based on socioeconomic status exist among income groups in the overall Canadian population (6-9). However, it is unknown how the health of low-income Canadians compares among the different provinces. The objective of this study was to investigate whether the province of residence, used as a proxy measure of social environment and adjusted for covariates, is related to the health of low-income Canadians. We sought to generate discussions on the provincial differences in social environments and to provide evidence for policy approaches to reducing health inequities in Canada that could also be generalized to other industrialized nations.

## Methods

### Data source

Data used in this study are from the 2005 Canadian Community Health Survey (CCHS) (12). A cross-sectional survey conducted by Statistics Canada, CCHS covers the population aged 12 years or older living in private households. Residents of Indian reserves, institutions, some remote areas, and military bases are not included. Participants provided their demographic, socioeconomic, behavioral, and health-related information. The survey response rate was 79%, yielding a sample of 132,947 respondents. A detailed description of the CCHS methodology is available (13).

We studied people who were 45 years or older, resided in 1 of the 10 provinces, and were from the lowest national income quintile. Income quintile was based on the national income distribution ratios, relative to the low-income cutoffs (14) derived from household income, number of family members, and community size. The 10 Canadian provinces studied are Newfoundland and Labrador, Prince Edward Island, Nova Scotia, New Brunswick, Quebec, Ontario, Manitoba, Saskatchewan, Alberta, and British Columbia. Forty-five years was chosen as the minimum age because chronic diseases commonly manifest in middle age. The final sample size was 14,475.

### Statistical methods

All estimates in this study were weighted to represent the entire population in each province for 2005. To account for the survey sampling design of the CCHS, we used the bootstrap technique (15-17) to calculate confidence intervals and coefficients of variation and to test the significance of differences between the estimates; significance was set at *P* < .05.

The health outcomes we considered were self-reported hypertension, diabetes, heart disease, cancer, mood disorder, and arthritis/rheumatism. Multivariate logistic regression models (18,19) were used to examine the relationship between each health outcome and province of residence. Analyses were adjusted for 3 demographic factors (age, sex, and immigration status) and 1 socioeconomic factor (education level). The bootstrap technique was used to test the significance of odds ratios and to estimate 95% confidence intervals. All behavioral factors (tobacco use, alcohol use, fruit and vegetable consumption, and physical activity) were excluded from the models, because they both influence health and result from socioeconomic factors, and thus are on the pathway from socioeconomic determinants to health outcomes.

## Results

When considering the entire population, British Columbia is the healthiest Canadian province in terms of both behaviors and health outcomes (Table 1). British Columbia residents have the longest life expectancy, a healthy lifestyle (highest prevalence of physical activity, lowest prevalences of smoking and obesity), and among the lowest prevalences of chronic diseases in the country.

Compared with British Columbia residents, those of most other provinces are more likely to report chronic diseases, especially hypertension, diabetes, and heart disease (Table 2); the Prairie provinces (Alberta, Manitoba, and Saskatchewan) are no different for diabetes and heart disease. In addition, British Columbia residents report a higher prevalence of mood disorder compared with residents of New Brunswick, Newfoundland and Labrador, Prince Edward Island, Quebec, and Saskatchewan.

When we consider only low-income populations, the health of British Columbia residents is no better than that of the other Canadian provinces. British Columbia loses its health advantage in diabetes to all provinces; hypertension to Alberta, Manitoba, Prince Edward Island, Quebec, and Saskatchewan; heart disease to Ontario, Prince Edward Island, and Quebec; and arthritis/rheumatism to Newfoundland and Labrador, New Brunswick, Manitoba and Saskatchewan. Compared with British Columbia, Quebec improves its position in mood disorder and arthritis/rheumatism and eliminates the gap in hypertension, diabetes, and heart disease. In fact, the health of low-income people from all 9 provinces is not much different from that of their British Columbia counterparts.

In the low-income population, we found that none of the other provinces is significantly better than Quebec for any of the major chronic conditions (Table 3). All other provinces are significantly worse than Quebec for at least 1 chronic disease studied. Compared with Quebec, the low-income population subset in British Columbia lost its health advantage for hypertension, diabetes, and heart disease. Manitoba, which is better off in diabetes and heart disease than Quebec when considering the entire population, is not when considering the low-income population. Similarly, the significant difference in favor of Saskatchewan over Quebec for heart disease also disappeared in the low-income subset.

To find out why Quebec is the healthiest province in Canada in terms of its low-income population, even though it is worse than British Columbia in terms of the broader population, we examined the social and behavioral factors for the low-income populations in both provinces. Most of the selected social and behavioral factors that contribute to chronic disease risk are significantly more prevalent among low-income residents of Quebec than among those of British Columbia, except for obesity and regular alcohol use, which were not significantly different (Table 4). The lower prevalence of social and behavioral risk factors in British Columbia compared with Quebec seems to contradict its higher prevalence of the selected chronic diseases.

Health outcomes depend on whether patients' health care needs can be met efficiently. We found no significant difference in percentage of overall population with unmet health care needs between Quebec (10.7%) and British Columbia (10.8%) (Table 5). However, when we examined low-income Quebec and British Columbia populations, we found that the percentage of people with unmet health care needs in British Columbia (15.6%) is significantly higher than that of their Quebec counterparts (9.5%). We further reviewed the major factors that distinguish the 2 provinces in this regard and found that 31.5% of British Columbia residents with unmet care needs reported cost as a factor, compared with only 6.4% of Quebec residents. Other factors, such as availability of care, do not seem to favor Quebec over British Columbia.

Ability to pay for health care for the low-income population depends to some degree on social assistance such as welfare. We found that low-income residents of Quebec are significantly more likely to report having welfare income (crude percentage, 16.9%) than are people from British Columbia (crude percentage, 11.0%). Adjusting for household income, number of people in the household, community size, age, and sex in a multivariate logistic regression model, we found that low-income residents of Quebec are more than twice as likely to report receiving welfare income as are residents of British Columbia.

## Discussion

Although British Columbia is the healthiest Canadian province overall, it is not the healthiest province for low-income people; Quebec is. This is true even though low-income British Columbians have better behavioral risk factor profiles and higher education levels than their Quebec counterparts. These findings point to the possible influence of social policy on health.

The health of low-income residents relies on the overall political, etiologic, and socioeconomic environment in which they live. Studies on associations between politics, social policy, and health outcomes (20,21) conclude that policies aimed at reducing social inequities, such as welfare state and labor market policies, appear to improve infant mortality rates and life expectancy at birth. Governments that build a comprehensive social environment with not only monetary support to low-income residents but also a systematic strategy based on full understanding of their health needs appear to have unique opportunities to demonstrate the extent to which health inequities can be eliminated.

Quebec is 1 of only 2 provinces with a comprehensive antipoverty strategy and is the only province that has enacted a law to combat poverty and social exclusion — Bill 112. The Collective for a Poverty-Free Quebec (22), an organization launched in May 2000, aims to progressively make Quebec, by 2013, one of the industrialized jurisdictions with the lowest poverty rates (23). Its efforts contributed to the development and eventual passage of Bill 112 in December 2002.

In Canada, each province has its own social support system with its own complex rules affecting type of assistance, eligibility for assistance, and rates of assistance. The jobs that low-income people have usually do not offer sick pay or extended medical service coverage. Some evidence exists that Quebec's antipoverty strategy, coupled with its unique social support system that includes a universal child care program, tax breaks and family benefits for parents with low-income jobs, and real estate tax refunds for low-income families, are beneficial for its low-income population (24). Quebec's antipoverty strategy and its enhanced social environment, formalized as law in 2002, may be responsible for the better health outcomes in Quebec's low-income population compared with British Columbia's.

Health insurance is also administered by each province separately and varies across the nation. Many medical services in Canada are not considered medically necessary and demand a full or partial fee, among them dental care, home care and senior care, prescription drugs, and prescription eyeglasses. No health insurance premium for children is required in Quebec, whereas in British Columbia, parents must pay premiums for themselves and their children. Additionally, Quebec is the only province that covers new drugs; elsewhere, they must be purchased out of pocket or through private drug plans that low-income people usually do not have. Quebec also has more health care resources. For example, in 2006 the number of specialist physicians was 106 per 100,000 population in Quebec, compared with 90 per 100,000 in British Columbia (25).

Our study has some limitations. Some vulnerable groups, such as Indian populations living on reserves and people without an address, were not reflected in the survey sample. Population surveys based on respondent recall may overestimate or underestimate diagnoses. Finally, the small sample size in the targeted population group limits the power to test for differences between individual provinces.

The chronic disease status of low-income populations varies considerably in Canadian provinces because of differences in behaviors, social policy, and possibly social environment. The right national antipoverty strategy could eliminate the effects of poverty on health. Reducing inequities in health outcomes through effective policy intervention in every Canadian province could also decrease the costs of chronic diseases to the health care system. The findings from this study provide evidence of a potential pathway from enhanced social policy to improved health outcomes for vulnerable populations.

## Figures and Tables

**Table 1:** Overall Health Status Based on Selected Self-Reported Health Conditions and Behaviors, Canadian Community Health Survey, 2005

Province	Years of Life Expectancy at Birth^b^	Prevalence of Conditions and Behaviors, %^c^			

		Any Chronic Condition	Daily Smoking	Active or Moderately Active	Obesity
Alberta	80.3	80.2	16.8	47.5^a^	18.4
British Columbia	81.2^a^	79.9^a^	11.8^a^	54.0*	15.5^a^
Manitoba	79.0	79.2*	14.1^a^	40.2	20.2
New Brunswick	79.8	82.7	16.1	38.5	23.5
Newfoundland and Labrador	78.2	84.0	16.1	38.2	24.7
Nova Scotia	79.3	85.2	15.9	40.4	22.4
Ontario	80.7^a^	83.2	14.5^a^	45.3^a^	17.9^a^
Prince Edward Island	79.8	85.7	18.4	31.1	22.2
Quebec	80.4^a^	78.4^a^	18.1	43.0	16.5^a^
Saskatchewan	79.3	82.7	18.1	41.5	23.0

a One of the 3 best-performing provinces in the category.

b Source: Statistics Canada (13).

c Among all residents aged 45 years or older. Conditions and behaviors were defined as follows: "any chronic condition," self-reported hypertension, diabetes, heart disease, cancer, mood disorder, and arthritis/rheumatism; "daily smoking," answer of "daily" to the question, "Do you smoke cigarettes daily, occasionally, or not at all?"; "active or moderately active," energy expenditure ≥1.5 kcal/kg/d based on self-report of activity in the past 3 months; "obesity," body mass index ≥30 kg/m^2^ based on self-reported height and weight (14).

**Table 2:** Odds of Selected Self-Reported Chronic Conditions in Canada for All Residents Versus British Columbia Residents, Canadian Community Health Survey, 2005^a^

**Province**	Hypertension	Diabetes	Heart Disease	Cancer	Mood Disorder	Arthritis/Rheumatism
**All residents**						
Alberta	1.16 (1.02-1.30)	1.08 (0.90-1.31)	1.07 (0.90-1.27)	0.87 (0.73-1.02)	0.97 (0.77-1.20)	1.08 (0.96-1.22)
British Columbia	1 [Reference]					
Manitoba	1.14 (1.00-1.31)	0.97 (0.80-1.17)	0.87 (0.71-1.07)	0.84 (0.70-1.01)	0.80 (0.61-1.05)	1.16 (1.02-1.31)
New Brunswick	1.65 (1.44-1.88)	1.47 (1.21-1.79)	1.70 (1.40-2.07)	0.94 (0.76-1.16)	0.77 (0.60-0.99)	1.20 (1.06-1.37)
Newfoundland and Labrador	1.68 (1.43-1.97)	1.72 (1.40-2.10)	1.33 (1.07-1.67)	0.73 (0.59-0.90)	0.67 (0.50-0.91)	1.43 (1.23-1.65)
Nova Scotia	1.48 (1.30-1.69)	1.59 (1.33-1.91)	1.84 (1.55-2.19)	1.22 (1.02-1.45)	1.01 (0.78-1.29)	1.42 (1.24-1.62)
Ontario	1.31 (1.19-1.43)	1.23 (1.08-1.40)	1.32 (1.16-1.49)	1.03 (0.92-1.14)	0.97 (0.85-1.11)	1.26 (1.15-1.38)
Prince Edward Island	1.34 (1.12-1.61)	1.48 (1.13-1.95)	1.78 (1.40-2.25)	0.92 (0.67-1.27)	0.61 (0.40-0.94)	1.51 (1.24-1.85)
Quebec	1.26 (1.15-1.39)	1.26 (1.10-1.45)	1.24 (1.10-1.40)	0.79 (0.69-0.90)	0.60 (0.50-0.72)	0.80 (0.72-0.88)
Saskatchewan	1.24 (1.10-1.39)	1.17 (0.97-1.41)	0.96 (0.80-1.17)	0.96 (0.81-1.12)	0.63 (0.50-0.78)	1.22 (1.07-1.39)
**Low-income residents**						
Alberta	1.26 (0.99-1.61)	0.96 (0.67-1.37)	1.04 (0.75-1.43)	0.88 (0.62-1.24)	0.89 (0.60-1.34)	1.06 (0.82-1.36)
British Columbia	1 [Reference]					
Manitoba	0.99 (0.74-1.32)	0.87 (0.61-1.24)	0.78 (0.55-1.12)	0.96 (0.66-1.39)	0.54 (0.34-0.88)	1.06 (0.81-1.39)
New Brunswick	1.61 (1.25-2.07)	1.09 (0.76-1.56)	1.68 (1.21-2.33)	0.99 (0.69-1.41)	0.68 (0.43-1.09)	1.13 (0.84-1.53)
Newfoundland and Labrador	1.74 (1.33-2.29)	1.36 (0.97-1.91)	1.41 (1.00-1.98)	0.74 (0.50-1.09)	0.75 (0.45-1.25)	1.25 (0.93-1.67)
Nova Scotia	1.79 (1.36-2.37)	1.29 (0.92-1.81)	1.37 (1.02-1.86)	1.22 (0.85-1.77)	0.58 (0.38-0.88)	1.36 (1.02-1.82)
Ontario	1.25 (1.04-1.50)	1.23 (0.94-1.62)	1.15 (0.91-1.44)	0.99 (0.75-1.30)	0.83 (0.63-1.10)	1.23 (1.01-1.50)
Prince Edward Island	1.29 (0.88-1.89)	1.41 (0.88-2.24)	1.25 (0.77-2.04)	1.13 (0.67-1.89)	0.33 (0.17-0.62)	1.76 (1.21-2.56)
Quebec	1.20 (0.99-1.45)	1.01 (0.77-1.33)	1.04 (0.83-1.31)	0.80 (0.59-1.07)	0.43 (0.32-0.59)	0.69 (0.56-0.85)
Saskatchewan	1.18 (0.92-1.51)	0.81 (0.58-1.13)	1.04 (0.77-1.42)	1.18 (0.83-1.66)	0.52 (0.34-0.80)	0.99 (0.76-1.28)

a Multivariate logistic regression was used to calculate all values, which are reported as odds ratios followed by 95% confidence intervals.

**Table 3:** Odds of Selected Self-Reported Chronic Conditions in Canada for All Residents Versus Quebec Residents, Canadian Community Health Survey, 2005^a^

**Province**	Hypertension	Diabetes	Heart Disease	Cancer	Mood Disorder	Arthritis/Rheumatism
**All residents**						
Alberta	0.92 (0.82-1.02)	0.86 (0.73-1.01)	0.86 (0.73-1.02)	1.09 (0.93-1.28)	1.60 (1.28-2.00)	1.35 (1.21-1.51)
British Columbia	0.79 (0.72-0.87)	0.79 (0.69-0.91)	0.81 (0.71-0.91)	1.26 (1.10-1.44)	1.66 (1.39-1.98)	1.25 (1.13-1.38)
Manitoba	0.90 (0.80-1.02)	0.76 (0.64-0.91)	0.70 (0.58-0.85)	1.07 (0.90-1.27)	1.33 (1.02-1.74)	1.45 (1.28-1.64)
New Brunswick	1.30 (1.15-1.48)	1.16 (0.97-1.39)	1.37 (1.15-1.63)	1.19 (0.97-1.45)	1.27 (0.99-1.63)	1.50 (1.33-1.70)
Newfoundland and Labrador	1.33 (1.15-1.54)	1.36 (1.12-1.64)	1.07 (0.86-1.33)	0.92 (0.75-1.13)	1.11 (0.83-1.49)	1.78 (1.55-2.05)
Nova Scotia	1.17 (1.04-1.32)	1.26 (1.07-1.48)	1.48 (1.25-1.76)	1.54 (1.30-1.82)	1.67 (1.30-2.14)	1.77 (1.58-1.99)
Ontario	1.03 (0.96-1.11)	0.98 (0.87-1.09)	1.06 (0.95-1.18)	1.30 (1.16-1.45)	1.61 (1.37-1.88)	1.57 (1.46-1.70)
Prince Edward Island	1.06 (0.89-1.27)	1.17 (0.90-1.52)	1.43 (1.14-1.80)	1.17 (0.86-1.60)	1.02 (0.66-1.57)	1.89 (1.55-2.30)
Quebec	1 [Reference]					
Saskatchewan	0.98 (0.88-1.09)	0.92 (0.77-1.10)	0.78 (0.65-0.93)	1.21 (1.03-1.41)	1.04 (0.83-1.30)	1.52 (1.35-1.71)
**Low-income residents**						
Alberta	1.05 (0.84-1.32)	0.95 (0.68-1.31)	0.99 (0.74-1.34)	1.10 (0.83-1.47)	2.07 (1.39-3.07)	1.53 (1.23-1.89)
British Columbia	0.83 (0.69-1.01)	0.99 (0.75-1.29)	0.96 (0.76-1.21)	1.26 (0.94-1.68)	2.32 (1.70-3.16)	1.44 (1.18-1.77)
Manitoba	0.82 (0.63-1.08)	0.86 (0.62-1.20)	0.75 (0.54-1.05)	1.20 (0.88-1.64)	1.26 (0.81-1.95)	1.53 (1.20-1.96)
New Brunswick	1.34 (1.07-1.68)	1.08 (0.77-1.49)	1.62 (1.19-2.19)	1.24 (0.93-1.65)	1.58 (1.03-2.42)	1.64 (1.27-2.11)
Newfoundland and Labrador	1.45 (1.13-1.86)	1.34 (0.99-1.81)	1.35 (0.98-1.86)	0.92 (0.66-1.30)	1.74 (1.07-2.83)	1.80 (1.39-2.33)
Nova Scotia	1.50 (1.16-1.93)	1.27 (0.94-1.72)	1.32 (1.01-1.72)	1.53 (1.12-2.10)	1.35 (0.92-1.99)	1.97 (1.57-2.48)
Ontario	1.05 (0.90-1.21)	1.22 (0.98-1.51)	1.10 (0.90-1.34)	1.24 (0.99-1.56)	1.93 (1.50-2.48)	1.78 (1.54-2.05)
Prince Edward Island	1.07 (0.74-1.56)	1.39 (0.90-2.15)	1.20 (0.75-1.93)	1.42 (0.88-2.29)	0.76 (0.40-1.45)	2.54 (1.79-3.62)
Quebec	1 [Reference]					
Saskatchewan	0.99 (0.80-1.21)	0.80 (0.60-1.06)	1.00 (0.78-1.29)	1.48 (1.11-1.97)	1.21 (0.78-1.86)	1.43 (1.14-1.78)

a Multivariate logistic regression was used to calculate all values, which are reported as odds ratios followed by 95% confidence intervals.

**Table 4:** Prevalence of Selected Negative Health Indicators Among Low-Income Residents^a^ of Quebec and British Columbia, Canadian Community Health Survey, 2005

**Indicator^b^**	Quebec, % (95% CI) n = 3,930	British Columbia, % (95% CI) n = 1,598	*P* Value^c^
Obesity	19.0 (17.1-21.0)	16.6 (13.5-19.6)	.18
Daily smoking	23.6 (21.4-25.9)	17.9 (15.0-20.8)	.003
Alcohol use	45.4 (42.9-48.0)	42.5 (38.6-46.3)	.22
Physical inactivity	17.4 (15.5-19.3)	13.2 (10.8-15.6)	.01
Poor education	52.5 (50.0-55.0)	31.8 (28.6-35.1)	<.001
Unattached individual	41.6 (39.2-44.0)	35.3 (32.2-38.3)	.002
Lack of home ownership	52.4 (49.9-54.9)	35.8 (32.3-39.4)	<.001
Nonimmigrant population	87.4 (85.2-89.6)	51.7 (48.0-55.4)	<.001
Unemployed	57.3 (54.6-59.9)	46.5 (42.8-50.2)	<.001

Abbreviation: CI, confidence interval.

a "Low-income" was defined as residents aged 45 years or older in the lowest income quintile. Source: Statistics Canada (16).

b Indicators were defined as follows: "obesity," body mass index ≥30 kg/m^2^ based on self-reported height and weight; "daily smoking," answer of "daily" to the question, "Do you smoke cigarettes daily, occasionally, or not at all?"; "alcohol use," drank alcoholic beverages at least once a month during the past 12 months; "physical inactivity," energy expenditure <1.5 kcal/kg/d based on self-report of activity in the past 3 months; "poor education," not receiving a high school diploma; "unattached individual," person living alone; "lack of home ownership," dwelling not owned by any member of household; "nonimmigrant population," did not move to Canada from a foreign country; and "unemployed," did not work for any length of time at a job or business in the last week.

c *P* values were calculated by using the bootstrap method.

**Table 5:** Prevalence of Unmet Health Care Needs Among Residents of Quebec and British Columbia, Canadian Community Health Survey, 2005

**Indicator**	Quebec, % (95% CI)	British Columbia, % (95%CI)	*P* Value
Unmet health care needs among all residents (Quebec n = 14,429; British Columbia n = 7,342)	10.7 (9.9-11.5)	10.8 (9.7-11.8)	.916
Unmet health care needs among low-income^a^ residents (Quebec n = 3,930; British Columbia n = 1,598)	9.5 (8.0-11.0)	15.6 (12.8-18.3)	<.001
Unmet needs due to costs^b^	6.4 (1.7-11.0)	31.5 (22.1-40.9)	<.001

Abbreviation: CI, confidence interval.

a "Low-income" was defined as residents aged 45 years or older in the lowest income quintile. Source: Statistics Canada (16).

b Among low-income residents with unmet health care needs.
